# Analysis of uveal melanomas and paired constitutional DNA for exclusion of a BAP1-tumor predisposition syndrome

**DOI:** 10.1007/s10689-022-00310-3

**Published:** 2022-08-03

**Authors:** Yasaman Arjmand Abbassi, Claudia Le Guin, Norbert Bornfeld, Nikolaos E. Bechrakis, Michael Zeschnigk, Dietmar R. Lohmann

**Affiliations:** 1 Institute of Human Genetics, University Hospital Essen, University Duisburg-Essen, Hufelandstr. 55, 45147 Essen, Germany; 2 Department of Ophthalmology, University Hospital Essen, University Duisburg-Essen, Hufelandstr. 55, 45147 Essen, Germany

**Keywords:** Uveal melanoma, *BAP1* gene, BAP1-TPDS, Predictive testing

## Abstract

Uveal melanoma (UM) is a rare tumor originating from melanocytic cells in the eye. Familial aggregation of UM is rare and can occur as part of the tumor predisposition syndrome BAP1-TPDS. However, family history alone will only identify a subset of patients with BAP1-TPDS. In the present study, we used sequential testing of tumor and blood DNA from UM patients for differential diagnosis of BAP1-TPDS. The study group was an unselected prospective cohort of patients from whom UM tissue was available. First, chromosome 3 status in tumor DNA was determined in all 140 patients who consented to participate. As tumors with disomy 3 rarely show BAP1 alterations, sequence analysis of this gene was performed in the 72 tumors with monosomy 3 (M3) or partial M3 only. We identified oncogenic BAP1 alterations in 52 of these tumors (72%). Targeted sequencing of DNA from matched peripheral blood showed pathogenic variants in two patients (3.8%) thus proving BAP1-TPDS. Only one of these two patients also had a medical history suggestive of this syndrome. Conversely, in three patients known to have had additional tumors before diagnosis of UM, constitutional heterozygosity for a *BAP*1 mutation was excluded. Altogether, in 50 patients we could exclude BAP1-TPDS with high diagnostic certainty. The results of our study support that genetic testing for BAP1-TPDS should be offered to all patients with UM. Moreover, as genetic information from the tumor can help exclude heritable risk, the strategy for analysis should include efforts to obtain tumor samples for testing.

## Introduction

Uveal melanoma (UM) is a malignant tumor that originates from melanocytic cells in the iris, ciliary body, and choroid of the eye [1]. UM is a rare tumor [1]—and, consequently, average individual risk is low. However, the incidence of UM shows marked differences worldwide with 1.3–8.6 cases per million per year in Europe and 0.2–0.3 cases per million in Africa and Asia [2]. This variation appears to be associated with differences in phenotypic features related to melanocyte functioning such as iris color, skin color, and ability to tan [3]. These phenotypic features are heritable traits, and in fact, some polymorphic genetic variants linked to pigmentation are associated with a risk to UM [4]. However, the strength of these associations is low, with odds ratios typically less than 2 [4]. Consequently, at the level of the individual, the effect of these polymorphic genetic variants on risk to UM has no clinical consequences.

The rare observations of families showing aggregation of patients with UM have suggested that heritable predisposition to UM can be transmitted as an autosomal dominant trait albeit with incomplete penetrance, i.e., absence of phenotype in some carriers [5]. The prototypical example of autosomal dominant tumor predisposition is heritable retinoblastoma [6]. The hypothesis that the development of this tumor depends on a two-step mutation process [7] has guided strategies that led to the identification of the *RB1* gene that is responsible for a heritable predisposition to retinoblastoma. Using a similar approach, the *BAP1* was identified as a likely candidate gene for a heritable predisposition to UM [8, 9]. The deubiquitinase BAP1 is multifunctional protein with tumor suppressor activity involved in chromatin remodeling, DNA damage response, cell cycle regulation, cell death, and differentiation [10, 11]. It turned out that genetic variants of *BAP1* can cause high risk not only to UM but also to a spectrum of other neoplasia [8, 9, 12, 13]. It is well established that heritable risk to UM can be part of a tumor predisposition syndrome with variable manifestation and incomplete penetrance (BAP1-tumor predisposition syndrome, BAP1-TPDS), and the number of families with known oncogenic variants of the *BAP1* gene has risen fast [14].

Families suspected to have BAP1-TPDS can benefit from genetic testing. Specifically, if diagnostic testing shows that a patient with UM has a BAP1-TPDS, this justifies repeated surveillance examinations for early detection of metachronous tumors, although details of the monitoring strategy need to be developed [15]. Identification of the family-specific pathogenic BAP1 gene variant facilitates predictive testing in relatives. In cancer predisposition syndromes with incomplete penetrance, relatives heterozygous for the mutation have an increased risk [16]. With predictive genetic testing, it is possible to exclude an increased risk in relatives. In addition to the benefit of psychological relief, overall health care cost is likely to be lower than without genetic testing [16].

Only a small proportion of patients with UM have a family history suggestive of BAP1-TPDS, i.e., most patients have isolated UM. Genetic testing has shown that some of these patients are heterozygous for *BAP1* variants that cause BAP1-TPDS [8, 9] and this finding establishes the diagnosis of BAP1-TPDS. However, if the genetic analysis is performed on DNA from blood only and fails to identify a pathogenic *BAP1* variant, then a BAP1-TPDS is less likely but not excluded. Because the spectrum of *BAP1* variants associated with BAP1-TPDS is very heterogeneous, it has to be expected that the false-negative rate of mutation screening tests is not negligible. In patients with isolated retinoblastoma, this problem is solved by mutation identification on DNA from the tumor first, followed by targeted testing in DNA from blood [17]. With this diagnostic strategy, it is possible to exclude an inherited predisposition in most patients with isolated unilateral retinoblastoma. The goal of the study presented here was to apply this strategy for differential diagnosis of BAP1-TPDS in patients with uveal melanoma.

## Material and methods

### Patients

We invited all UM patients treated in our clinic between October 2014 and October 2016 and from whom tumor material was available to participate in the study. From these, 140 patients agreed. Tumor samples were obtained either by biopsy sampling, by enucleation or endoresection, depending on treatment of the primary tumor. Chromosome 3 status (disomy 3, or monosomy 3) of all tumor samples was analyzed by microsatellite analysis (MSA). *BAP1* sequencing was performed on DNA from all samples with monosomy 3 (M3) or partial losses of chromosome 3 (partM3) (n = 72).

### Samples and genetic analysis

Genomic DNA was isolated from enucleated tumors by the phenol–chloroform method as described previously [18]. DNA from biopsy samples was isolated using the QIAamp DNA Mini Kit (Qiagen, Hilden, Germany). DNA from blood was isolated using the FlexiGene kit (Qiagen). The chromosome 3 status of all tumor samples was determined by STR-genotyping (microsatellite analysis, MSA) using eight chromosome 3 markers as described elsewhere [19]. To detect tumors with isodisomy 3 all tumors showing loss of heterozygosity (LOH) of chromosome 3 markers by MSA were subjected to chromosome 3 MLPA (MRC Holland, probemix P027-C2). *BAP1* mutation screening was performed by Sanger sequencing in DNA from 72 UM with M3 or partM3 as described in Martin et al., [20]. In samples without clear results by Sanger sequencing, NGS panel sequencing was performed. Copy number analysis of all *BAP1* exons was determined by Multiplex ligation-dependent probe amplification (MLPA) using SALSA MLPA probemix P417-B2 BAP1 (MRC Holland, Amsterdam, The Netherlands) following the manufacturer's instructions.

### Panel sequencing

In this study, Agilent SureSelect^XT HS^ target enrichment system was used to enrich the genomic regions of interest. The customized panel was designed to capture all coding regions of *BAP1*, *SF3B1*, *EIF1AX*, *CYSLTR2, PCLB4, RB1,* and exons 4 and 5 of the *GNAQ* and *GNA11* genes. About 100 ng genomic tumor DNA was subjected to library preparation according to the manufacturer's instructions (protocol version A1, July 2017). Briefly, genomic DNA was fragmented using a Covaris S220 to an average size of 150 to 200 bp, and the fragments were ligated to molecular barcodes. The captured fragments enriched for the target sequences were sequenced on Illumina MiSeq by paired-end sequencing with 2 × 150 bp. We obtained a coverage of > 400 × for all coding regions in each sample. For mapping of the reads and mutation calling, the fastq files were analyzed using SureCall (version 4.1.1.5, Agilent). Sanger sequencing was used to re-sequence regions showing mutations by Panel Sequencing in tumor DNA and to sequence the respective blood DNA of the patient. The pathogenicity of the germline variants was determined using InterVar (https://wintervar.wglab.org/) which is based on the ACMG/AMP 2015 guidelines [21]. For evaluating the oncogenicity of somatic variants we have adhered to the applicable criteria of the ACMG guidelines: Nonsense, frameshift, exon skipping and deletion variants in conjunction with LOH of *BAP1* are classified as oncogenic mutations. Missense variations in *BAP1* with a ClinVar or COSMIC entry are classified as oncogenic if in addition computational data indicate impaired protein function.

## Results

### Participants

All patients (n = 265) who requested prognostic biomarker testing (chromosome 3 status in tumor DNA) were eligible (148 males (55.8%), median age = 61.3 years, interquartile range (IQR) = 15.8 years and 117 female, median age = 61.7 years, IQR = 21.2 years). Of these, 140 (52.8%) consented to participate in this study (83 males (59.3%) with median age at diagnosis = 60.8 years and IQR = 14 years; 57 females with median age at diagnosis = 57.9 years and IQR = 20.1 years).

### Chromosome 3 status

**Fig. 1:**
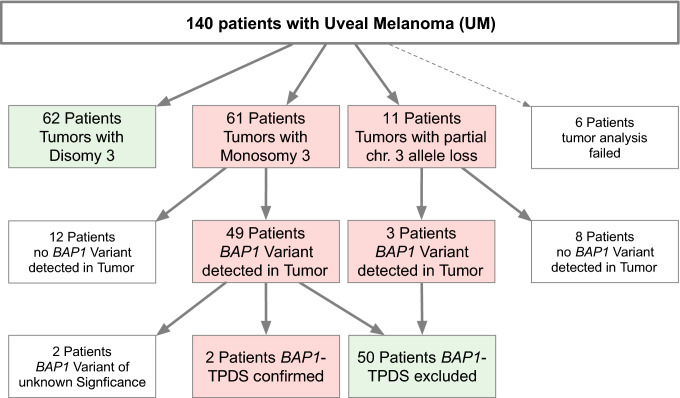
Overview of the study cohort and the grouping of patients depending on the chromosome 3 status and the mutation status of the primary tumor

Mutational inactivation of the *BAP1* gene is rarely seen in the class of UMs with disomy 3 (D3) if isodisomy 3 is excluded [22, 23]. To focus the analytical efforts on samples likely to have *BAP1* gene alterations we determined the chromosome 3 status in tumors before further mutation analysis. Genotype data of polymorphic STR-loci from 134 patients met our technical quality criteria for determining the chromosome 3 status. These criteria require that a minimum number of loci are heterozygous in DNA from blood. Uveal melanomas from 62 patients (46%) showed no loss of heterozygosity at any informative locus and, therefore, were classified as disomy 3 tumors (Fig. 1). In tumors from 61 patients (46%), monosomy 3 was diagnosed because of loss of heterozygosity at all informative loci (Fig. 1). In UMs from the remaining 11 patients (6%), only some of the informative loci on chromosome 3 showed allele loss (Fig. 1). This pattern of allele loss is typical for uveal melanomas with deletions only of parts of chromosome 3 (partM3).

### Mutation analysis of the BAP1 gene in uveal melanoma

BAP1 mutation analysis was performed in UM with monosomy 3 or partial deletions of chromosome 3 (72 tumors). Sanger sequencing was chosen as the first-line method of sequencing analysis. Those samples that did not meet the quality criteria for mutation analysis, which require, among other parameters, that sequence data has been obtained for all coding and invariant splice site regions of the *BAP1* gene, were subjected to panel sequencing which included the *BAP1* gene. Copy number variant detection by MLPA was performed on samples that did not show a oncogenic small variant. Alterations of the *BAP1* gene were identified in 52 of the 72 tumors with M3 or partM3 (72%). In the tumors with partM3 the percentage of samples with *BAP1* mutation was lower (27%) than in the M3 group.

In 50 of 52 tumors we detected oncogenic *BAP1* alterations (Table 1). Alterations identified in these 50 tumors included two deletions that spanned one or more exons. The remaining 48 alterations were small variants. In these tumors the signal of the normal allele was low compared to that of the variant allele. This result is in line with hemizygosity at the *BAP1* locus, which is expected in tumors with monosomy 3. In tumors with alterations only of parts of chromosome 3 this result suggests that the BAP1 locus is within regions of allele loss.

**Table 1:** BAP1 sequence variants in 52 uveal melanomas

ID	Chr.3 status	BAP1 variant	HGVS_p	Functional description	origin	Seq. method	Mutation ID ClinVar	ClinVar recurrences	Mutation ID cosmic	COSMIC recurrences
TP1	M3	exon 5 deletion	p.(Leu86_Glu125del)	In-frame-deletion	Somatic	Panel	NA	–	NA	–
TP2	M3	exon 8–10 deletion	p.(Gly194_Asp311)	In-frame-deletion	Somatic	Sanger	NA	–	NA	–
TP3	M3	c.1984-19_1984-4del	p.(Ile662Alafs*5)	Exon-skipping fs	Somatic	Sanger	NA	–	NA	–
TP4	M3	c.784-2A > G	p.(Leu262Metfs*22)	Exon-skipping fs	Somatic	Sanger	NA	–	NA	–
TP5	PartM3pq	c.122 + 3_122 + 11del	p.(Gly23Alafs*23)	Exon-skipping fs	Somatic	Sanger	NA	–	NA	–
TP6	M3	c.1729G > C	p.(Tyr418Glyfs*64)	Exon-skipping fs	Somatic	Sanger	114,540,411	1	COSV56239857	1
TP7	M3	c.1984-2A > C	p.(Ile662Alafs*5)	Exon-skipping fs	Germ line	Sanger	NA	–	NA	–
TP8	M3	c.67 + 13_67 + 53del	p.(Gly13Alafs*29)	Exon-skipping fs	Somatic	Sanger	NA	–	NA	–
TP9	M3	c.376-5_376-2del	p.(Ser126Alafs*6)	Exon-skipping fs	Somatic	Sanger	NA	–	NA	–
TP10	M3	c.540_580 + 20delinsC	p.(Pro147Alafs*46)	Exon-skipping fs	Somatic	Panel	NA	–	NA	–
TP11	M3	c.363_375 + 41delTTT	p.(Leu86_Glu125del)	In-frame-deletion	Somatic	Panel	NA	–	NA	–
TP12	M3	c.256-12_310del	p.(Leu86_Glu125del)	Exon-skipping in-frame	Somatic	Sanger	NA	–	NA	–
TP13	M3	c.1049_1050delinsT	p.(Pro350Leufs*12)	Frameshift	Somatic	Sanger	NA	–	NA	–
TP14	M3	c.39_42del	p.(Leu14Serfs*57)	Frameshift	Somatic	Sanger	NA	–	NA	–
TP15	M3	c.500_511del	p.(Ser10_Gly13delfs*67)	Frameshift	Somatic	Sanger	NA	–	NA	–
TP16	PartM3p	c.1654dup	p.(Asp552Glyfs*15)	Frameshift	Somatic	Sanger	NA	–	NA	–
TP17	M3	c.577del	p.(His193Metfs*38)	Frameshift	Somatic	Sanger	NA	–	COSV105168035	1
TP18	M3	c.740del	p.(Val247Glyfs*2)	Frameshift	Somatic	Sanger	NA	–	NA	–
TP19	M3	c.1366del	p.(Gln456Argfs*115)	Frameshift	Somatic	Sanger	NA	–	NA	–
TP20	M3	c.400del	p.(Ala134Profs*53)	Frameshift	Somatic	Sanger	NA	–	NA	–
TP21	PartM3q	c.585del	p.(Trp196Glyfs*35)	Frameshift	Somatic	Sanger	NA	–	NA	–
TP22	M3	c.843_846delinsTA	p.(Pro282Argfs*24)	Frameshift	Somatic	Sanger	NA	–	NA	–
TP23	M3	c.26_47del	p.(Glu9Alafs*56)	Frameshift	Somatic	Sanger	NA	–	NA	–
TP24	M3	c.376_377del	p.(Ser126Glnfs*16)	Frameshift	Germ line	Sanger	NA	–	NA	–
TP25	M3	c.350_375 + 45del	p.(Asp117_Glu126fs*142)	Frameshift	Somatic	Sanger	NA	–	NA	–
TP26	M3	c.1965_1966del	p.(Lys656Glufs*7)	Frameshift	Somatic	Sanger	114,534,267	1	NA	–
TP27	M3	c.170_179del	p.(Arg57Glnfs*12)	Frameshift	Somatic	Sanger	NA	–	NA	–
TP28	M3	c.880_899del	p.(Leu294Glyfs*6)	Frameshift	Somatic	Sanger	NA	–	NA	–
TP29	M3	c.570_580del	p.(Ile191Alafs*48)	Frameshift	Somatic	Sanger	NA	–	NA	–
TP30	M3	c.1762_1796delinsA	p.(Pro588Argfs*18)	Frameshift	Somatic	Sanger	NA	–	NA	–
TP31	M3	c.1636del	p.(Tyr546Thrfs*25)	Frameshift	Somatic	Sanger	NA	–	NA	–
TP32	M3	c.522_530del	p.(Pro175_Thr177)del	In-frame	Somatic	Sanger	NA	–	NA	–
TP33	M3	c.182_200delinsC	p.(Lys61_Asp67delinsThr)	In-frame	Somatic	Sanger	NA	–	NA	–
TP34	M3	c.520_525del	p.(Val174_Pro175del)	In-frame	Somatic	Sanger	NA	–	NA	–
TP35	M3	c.692_706del	p.(Met231_delinsAsp236 Asn)	In-frame	Somatic	Sanger	NA	–	NA	–
TP36	M3	c.486_507del	p.(Thr164_Phe170del)	In-frame	Somatic	Sanger	NA	–	NA	–
TP37	M3	c.535C > T	p.(Arg179Trp)	Missense	Somatic	Sanger	NA	–	COSV56230719	4
TP38	M3	c.299 T > G	p.(Leu100Arg)	Missense	Somatic	Panel	NA	–	COSV56235221	1
TP39	M3	c.188C > G	p.(Ser63Cys)	Missense	Somatic	Sanger	114,533,374	5	COSV56230632	1
TP40	M3	c.505C > T	p.(His169Tyr)	Missense	Somatic	Sanger	114,535,404	5	COSV56233385	5
TP41	M3	c.518A > C	p.(Tyr173Ser)	Missense	Somatic	Sanger	114,538,920	2	COSV56238100	2
TP42	M3	c.509 T > C	(p.Phe170Ser)	Missense	Somatic	Sanger	NA	–	NA	no
TP43	M3	c.92A > G	p.(Glu31Gly)	Missense	Somatic	Sanger	114,541,608	1	COSV56241651	2
TP44	M3	c.422A > G	p.(His141Arg)	Missense	Somatic	Sanger	114,532,971	3	COSV56229904	3
TP45	M3	c.188C > G	p.(Ser63Cys)	Missense	Somatic	Sanger	114,533,374	5	COSV56230632	1
TP46	M3	c.272G > A	p.(Cys91Tyr)	Missense	Somatic	Sanger	114,541,032	2	COSV56240775	2
TP47	M3	c.284C > G	p.(Ser98Arg)	Missense	Somatic	Sanger	NA	–	NA	–
TP48	M3	c.588G > A	p.(Trp196*)	Nonsense	Somatic	Sanger	114,534,223	4	COSV56231737	4
TP49	M3	c.588G > A	p.(Trp196*)	Nonsense	Somatic	Sanger	114,534,223	4	COSV56231737	4
TP50	M3	c.1447C > T	p.(Gln483*)	Nonsense	Somatic	Sanger	114,536,409	1	NA	–
TP51	M3	c.2057-4G > T	exon 17 splice acceptor	No effect	Germ line	Panel	NA	–	NA	–
TP52	M3	c.2057-4G > T	exon 17 splice acceptor	No effect	Germ line	Sanger	NA	–	NA	–

Mutation description refers to BAP1 transcript reference sequence NM_004656.3. In both tumors with partial loss of 3p regions, *BAP1* gene is included in deleted region. With the exception of the variant indentified in TP7 all missense mutations are classified as oncogenic according to ACMG criteria as applicable

Two of 52 tumors did not show a bona fide pathogenic alteration but an identical single base substitution at the -4 position of the splice donor site of exon 17. This position is not part of the invariant splice donor sequence motive and the base substitution does not create an alternative donor site. In silico analysis of this intronic variant have previously shown that it is not expected to affect splicing [24]. In both patients, this variant allele was also present in the constitutional DNA and, therefore, it is plausible that this alteration is a rare and likely neutral polymorphic variant.

### Analysis of DNA from blood for patient-specific variant BAP1 alleles

We performed *BAP1* mutation analysis targeting the oncogenic variants detected in the tumor on DNA from matched peripheral blood (50 patients). In 48 patients (96%) results showed that the oncogenic variant identified in the tumor resulted from a somatic mutation in the patient. Two patients (4%) showed heterozygosity for the oncogenic variant identified in the tumor, thus indicating a germ-line origin (TP7, TP24). Both variants were classified as pathogenic by “InterVar” based on ACMG criteria. One of the germline mutations p.(Ser126Glnfs*16) has been identified in a patient with mesothelioma, previously [25]. As we had no access to constitutional DNA from parents or other relatives of these two patients, we could not track the origin of the variants any further.

### Functional types and localization of oncogenic alterations

**Fig. 2:**
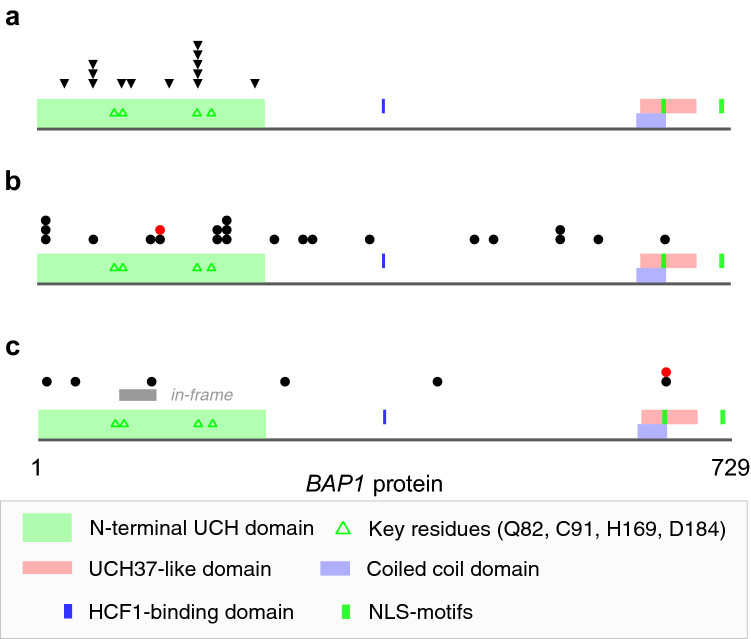
Map of *BAP1* oncogenic sequence variants in 50 uveal melanomas. **a** Black triangles: location of somatic missense or in-frame variants. **b** and **c** Black circles: location of premature stop codons due to nonsense, frameshift or splice variants caused by somatic alterations. Red circles: location of premature stop codons due to nonsense, frameshift or splice variants caused by constitutional alterations. Grey bar: in-frame loss of codons due to splice variant caused by somatic alteration.

Missense and in-frame length variants (11 and 5, respectively) were identified in samples from 16 patients, and all were of somatic origin (Table 1). Amino acids predicted to be altered by these mutations were all located in the N-terminal UCH domain of the BAP1 protein (Fig. 2a). One missense variant, c.188C > G [p.(Ser63Cys)], was identified in tumors of two patients and is also listed as a recurrent somatic variant (n = 5) in the Catalogue Of Somatic Mutations In Cancer (COSMIC V90_38_MUTANTCENSUS). Of the other 14 variants predicted to alter only single or few amino acids of the BAP1 gene 9 variants (56%) are also listed in COSMIC. The interpretation of one missense mutation p.(Ser98Arg) although located in the UCH domain and present on the background of chromosome 3 loss remains uncertain as computational data do not indicate a severe impairment of protein function.

Tumors from 32 patients had variants resulting in premature termination codons (3 nonsense, 19 frame-shift, and ten splice site variants; Table 1). The locations of these terminations showed no clustering to particular domains of the BAP1 protein (Fig. 2b). No premature termination was located within the last exon and the 50 bp 3′-parts of the penultimate exon of the *BAP1*, which are regions expected not to elicit nonsense-mediated decay [26]. One of the variants resulting in premature termination codons, c.588G > A [p.(Trp196Ter)], was identified in tumors of two patients and is also listed as a recurrent somatic variant (n = 4) in COSMIC. However, in all, only 4 of the 28 (14%) premature termination variants identified here are also listed in COSMIC.

One tumor (TP1) showed a deletion of the splice acceptor-site of intron 5 and parts of exon 5 (Table 1). The expected consequence of this variant is a loss of exon 5 from the mRNA and, on translation, an in-frame deletion of Leu86 (CTG) to Glu125(GAG) (Fig. 2c). These amino acids are part of the N-terminal UCH domain of the BAP1 protein.

### Relations between constitutional BAP1 genotype and phenotypic features

**Fig. 3:**
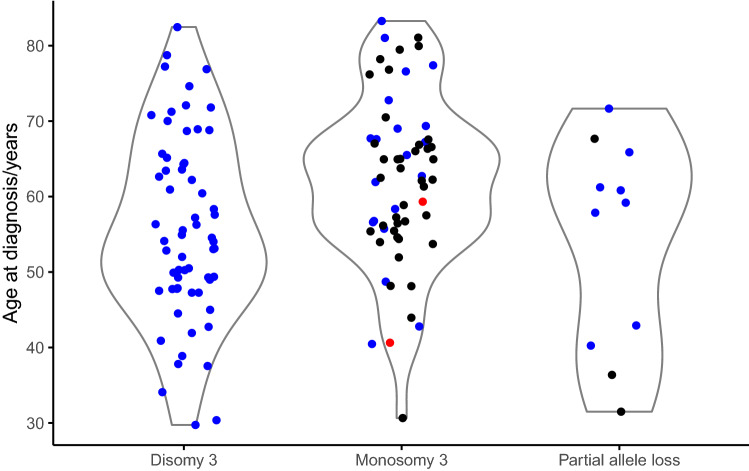
Density plots of age at diagnosis of 140 patients included in the study grouped by chromosome 3 status

The ages at diagnosis of the two patients with pathogenic *BAP1* germ-line variants ranked at positions 4 and 21 within the group of 50 patients with *BAP1* variants. Their age rank positions within the whole group of 140 patients were similar (positions 12 and 71 of 140). In the density plots of age at diagnosis, the two patients with germ-line variants also do not appear to be outliers (Fig. 3).

At the time of diagnosis of the primary tumor, data on medical history concerning tumors outside of the eye was available from 100 patients. Two patients had a history of renal cell cancer diagnosed prior to uveal melanoma but only one of these two patients was heterozygous for a pathogenic *BAP1* germ-line mutation. This patient also had a history of cutaneous malignant melanoma. In the two other patients with diagnosis of cutaneous malignant melanoma prior to uveal melanoma, a common pathogenic *BAP1* gene germline variant as root cause is highly unlikely because the *BAP1* gene mutations that we identified in their tumors were not present in DNA from blood and, therefore, of somatic origin. However, as we cannot exclude low-dose mosaicism and thus it is conceivable that tumors can originate from cells of a mutant sector that share a oncogenic *BAP1* gene somatic variant. Data on family history was incomplete for most patients. Of note, the father of a *BAP1* heterozygous patient in our study had died from pleural mesothelioma, a tumor type that is very rare in the general population but relatively frequent in families with BAP1-TPDS (for review see [14]).

### Discussion

In this study we used genetic testing of tumors for molecular differential diagnosis of BAP1-TPDS in patients with UM. We adapted a stepwise testing strategy similar to the one that is used for routine in genetic testing in retinoblastoma to the requirements specific for uveal melanoma. The initial analysis step identifies tumors with complete or partial monosomy 3 and, in the next step, DNA from these tumors is analyzed for *BAP1* gene alterations. With decreasing sequencing costs due to NGS, we would suggest sequencing of tumor and blood tissue simultaneously instead of one after another which is more time efficient. A BAP1-TPDS is excluded in patients in whom a somatic origin of these alterations is established by analysis of constitutional DNA. With this three-step strategy, we could safely exclude a BAP1-TPDS in 50 of 72 (70%) patients with a M3 or partM3 UM. Since germ-line variant alleles of the BAP1 gene are rare in UM patients with D3 tumors (odds ratio 0.12, derived from data published by Ewens 2018 [22]), TPDS is also unlikely in the group of 62 patients with D3 tumors. However, it remains to be shown if extending the diagnostic strategy to patients with UM with D3 leads to detection of further BAP1-TPDS with a frequency higher than those in the normal (UM-free) population [22].

Most of the alterations with confirmed somatic origin resulted in premature termination outside of the regions that are expected to evade nonsense mediated mRNA decay [26] and, therefore, are expected to result in loss of function, which is typical for the spectrum of *BAP1* alterations in cancer. All amino acids altered by missense substitution or short in-frame losses belonged to the ubiquitin C-terminal hydrolase (UCH) domain of the *BAP1* protein. This catalytic domain plays a key role by mediating deubiquitination of chromatin elements such as H2A [27]. Most of the missense substitutions identified here have been reported as somatic mutations in other tumors and this also supports that these alterations can be classified as pathogenic. Recurrence of somatic missense mutations was also found in a recent report by Ewens et al. [22]. Thus, it appears that the set of oncogenic missense mutations of the BAP1 gene is smaller than the set of variants that result in premature termination.

We did not identify oncogenic *BAP1* alterations in DNA from 14 to 8 tumors (29%) with M3 or partM3, respectively. In the tumors with partM3 the percentage of samples with *BAP1* mutation was much lower (27%) compared to M3 tumors which is in concordance with the observation that most of these tumors show a mutation pattern typical for the UM with D3 [20]. Between reported studies the proportion of tumors with M3 with a oncogenic *BAP1* alteration varies and the detection rate in our study is well within this range [23, 28]. It is conceivable that some tumors with M3 may have *BAP1* gene alterations that are not within the scope of the detection methods used here. Specifically, we expect an improved rate of detection if sequencing libraries cover the complete *BAP1* gene region instead of focusing enrichment of the exons only. The need for extension of the scope of analysis by NGS has already been pointed out by Field et al. 2018 [23, 28]. Another advantage of NGS-based analysis is a lower detection limit compared to Sanger sequencing. This allows the detection of sequence variants in heterogeneous tumor tissue containing high proportion of normal cells [23] thus increasing the proportion of fully informative cases, although it does not lead to the discovery of additional heritable cases.

In 2 patients (4% of 50 patients with *BAP1* mutant tumors, 1.4% of all 140 patients) we determined heterozygosity for pathogenic *BAP1* gene variants and this established the diagnosis of a BAP1-TPDS. Both variants are expected to result in premature termination which is the predominant type of consequence of pathogenic *BAP1* alterations. Premature termination was also the most frequent biological consequence among the *BAP1* germ-line variants reported by Ewens et al. [22]. By contrast, studies restricted to mutation analysis on DNA from blood tend to report a higher proportion of missense type variants and these include a high proportion of variants of uncertain pathogenic significance (VUS) [22]. In fact, 608 of 738 (82%) gene variants in the *BAP1* coding region listed in ClinVar in the context of heritable tumor susceptibility are VUS (https://www.ncbi.nlm.nih.gov/clinvar, query of 14.7.2021). Using our strategy, which requires initial sequencing of tumor DNA, only half as much VUS are expected due to loss of one *BAP1* allele in most tumors. In other words, results of mutation analysis on DNA from blood only are often insufficient to provide the evidence needed to demonstrate or exclude a BAP1-TPDS with high confidence. In vitro assays that assess if some functions of BAP1 protein are altered depending on certain alterations can provide additional data in support of the pathogenicity of exonic *BAP1* variants [29, 30]. However, we agree that “further research into BAP1 functions is needed” to arrive at “a consensus on mandatory experimental assays to assess VUS” [30].

Only one of the two patients that we identified to be heterozygous for a pathogenic *BAP1* alteration was also positive for features proposed as referral guidelines for genetic diagnostics of a BAP1-TPDS [31]. This patient was diagnosed with CMM and RCC (at age 28 and 39 years, respectively) prior to the diagnosis of UM (at age 59 years). Moreover, one parent of this patient had died of pleural mesothelioma, a rare cancer that is frequent in patients with a BAP1-TPDS. The medical and family history of the other BAP1-TPDS patient identified in our study did not meet the referral criteria proposed by Chau et al. [31]. However, with a relatively young age at diagnosis of UM (40 years) this patient qualifies according to more relaxed guidelines [9]. From recent studies it appears that population-based analyses, which avoid the ascertainment biases introduced by selection of high-risk patients, are required to obtain a better understanding of the BAP1-TPDS [22]. In other cancer predisposition syndromes such as hereditable breast and ovarian cancer (HBOC) it has turned out that initial estimates of penetrance, which were derived from observations of familial aggregation of cancer cases, were inflated. Unbiased data are required to model the cancer risk of individuals who are heterozygous for a pathogenic *BAP1* allele. Such a statistical model is essential to derive surveillance plans for early detection. The results of our study support that genetic diagnosis of a BAP1-TPDS should be offered to all patients with UM. Moreover, the testing strategy should include efforts to obtain genetic information from the tumor.

## Data Availability

Not applicable.
